# Role of RNA Oxidation in Neurodegenerative Diseases

**DOI:** 10.3390/ijms21145022

**Published:** 2020-07-16

**Authors:** Ziqian Liu, Xiatian Chen, Zhe Li, Wei Ye, Hongyan Ding, Peifeng Li, Lynn Htet Htet Aung

**Affiliations:** 1 Center for Molecular Genetics, Institute for Translational Medicine, Qingdao University, Qingdao 266000, China; liuziqian96@163.com (Z.L.); chenxiatian1216@163.com (X.C.); m17852020797@163.com (Z.L.); 2 School of Basic Medicine, Qingdao University, Qingdao 266000, China; 3 Jiangsu Provincial Engineering Research Center for Biomedical Materials and Advanced Medical Device, Huaiyin Institute of Technology, Huaian 223003, China; weiye@hyit.edu.cn

**Keywords:** RNA oxidation, oxidative damage, neurodegenerative disorders, treatment strategies

## Abstract

In the history of nucleic acid research, DNA has always been the main research focus. After the sketch of the human genome was completed in 2000, RNA has been started to gain more attention due to its abundancies in the cell and its essential role in cellular physiology and pathologies. Recent studies have shown that RNAs are susceptible to oxidative damage and oxidized RNA is able to break the RNA strand, and affect the protein synthesis, which can lead to cell degradation and cell death. Studies have shown that RNA oxidation is one of the early events in the formation and development of neurodegenerative disorders, including Alzheimer’s disease, Parkinson’s disease, and amyotrophic lateral sclerosis. However, its molecular mechanism, as well as its impact on these diseases, are still unclear. In this article, we review the different types of RNA oxidative damage and the neurodegenerative diseases that are reported to be associated with RNA oxidative damage. In addition, we discuss recent findings on the association between RNA oxidative damage and the development of neurodegenerative diseases, which will have great significance for the development of novel strategies for the prevention and treatment of these diseases.

## 1. Introduction

RNA is known to be essential for all living cells and performs many other functions besides protein synthesis. Contrary to DNA damage studies, RNA damage has only recently been concerned [1]. Although the RNA is only encoded by a small part of the genome in higher organisms, research shows that a great majority of the genome is transcribed, which indicates that the function of a large amount of RNA has not been revealed [2]. In the cell, RNA accounts for 80% to 90% of the total cellular nucleic acid and is more abundant than DNA. Therefore, RNA can be a principal target for nucleic acid damage agents. RNA damage may affect cells as a result of any changes in RNA function. Many factors like ultraviolet light, reactive oxygen species, and nitrogen (ROS and RNS) can induce oxidative damage in RNA. Intracellular RNAs suffer from the same oxidative damage as other biological macromolecules such as DNA. RNA damage may have serious adverse effects on RNA chain, protein synthesis, and cell function. RNA is mainly single-stranded and its bases are not protected by hydrogen bonds or specific proteins, so it is more susceptible to oxidative damage than DNA [3]. Therefore, oxidative damage of protein-coding RNA or non-coding RNA can affect the gene expression. Although RNA oxidation is not as fatal as genomic mutations, RNA oxidative damage is a typical feature of neuronal fragility, suggesting that RNA oxidation may promote the occurrence of chronic degeneration [4,5], including Alzheimer’s disease (AD), Parkinson’s disease (PD), Lewy body dementia, and prion diseases [6]. Increasing research evidence that preventing damaged nucleotides play a role in translation can significantly reduce the harmful effects of oxidative RNA on protein synthesis. An in-depth study on the molecular mechanisms involved in oxidative RNA damage may provide important implications for the pathogenesis and treatment strategies of neurodegenerative disorders and other diseases [7,8]. 

## 2. RNA Oxidation

Because the structure of ribonucleic acid is similar to deoxyribonucleic acid, the nucleotide structures of RNA and DNA are still similar after oxidation modification, especially guanosine. 8-oxidized guanosine is the most studied oxidized RNA damage marker at present. 8-oxidized guanosine exists widely in various tissues and body fluids (such as the brain, spinal cord, liver, artery, urine, cerebrospinal fluid, etc.) [9,10,11]. Reactive oxygen species (ROS), ultraviolet rays (UV), and alkylating agents can cause nucleic acid cross-linking, nucleic acid strand breakage, and base modification damage and other RNA oxidative damage under physiological or pathological conditions, of which the most important factor is ROS [12]. Some factors in the body, such as abnormal mitochondrial function, usually increase the number of ROS, and certain physiological reactions also produce ROS. Studies have already revealed that ROS not only play a cytotoxic role directly but also indirectly regulate the expression of some genes by affecting the cellular signal transduction system and interact with ribose, bases, nucleotides, and oligonucleotides [13]. ROS induce chemical reactions, which lead to the oxidative modification of RNA.

Due to the following four reasons, the brain is more susceptible to oxidative damage. (1) High oxygen consumption of the brain: the oxygen consumption of the human brain accounts for 20–25% of the total oxygen consumption of the human body [14]. (2) The brain is prone to high levels of polyunsaturated fatty acids [14]. (3) High levels of transition metals in the brain can catalyze the reaction that produces ROS [15]. (4) Compared with other organs, the brain is relatively lacking in antioxidant enzymes: for example, the content of catalase in the brain is only 10–20% of the liver and heart [16]. At this time, superoxide (^·−^O_2_), hydrogen peroxide (H_2_O_2_), and the hydroxyl radical (^·^OH) produced by the normal mitochondrial electron transport chain through normal cell metabolism will threaten neurons, leaving the neurons of the brain continuously exposed to ROS [14,15,16]. The ^·^OH can only diffuse a few nanometers in the tissue [17] and ^·−^O_2_ can hardly penetrate cell membrane [18]. When the level of ROS in the cell exceeds the defense ability of the cell, the RNA widely present in the cytoplasm is continuously in contact with the ROS and is attacked by the ROS, causing oxidative damage to the RNA. In general, we do not pay much attention to the cause of RNA damage because maybe that damaged molecules will not accumulate due to the transient nature of RNA. However, the damage caused by ROS occurs within a few minutes, and the average half-life of most people’s mRNA can last for 10 hours [1]. In addition, stable RNA species (mainly rRNA and tRNA) constitute the majority of cellular RNA and will not degrade during exponential growth [19]. Therefore, the damaged RNA will accumulate in the cell and pose a challenging problem for the cell. In the brain of AD patients, dysfunctional mitochondria may produce large amounts of ROS [20], and 8-oxo-guanosine (8-oxoGuo) levels were detected in the brain of AD patients after death [21]. All of these suggest that the formation of ROS that causes RNA oxidation in AD is likely to be the cause of mitochondrial abnormalities. Interestingly, the phenomenon of mitochondrial abnormalities [22] is also found in the substantia nigra of PD, which makes this mechanism a common theme in the cascade of neurodegenerative diseases [23].

## 3. Mechanism of RNA Oxidation 

### 3.1. Structural Changes in Oxidized RNAs

**Figure 1:**
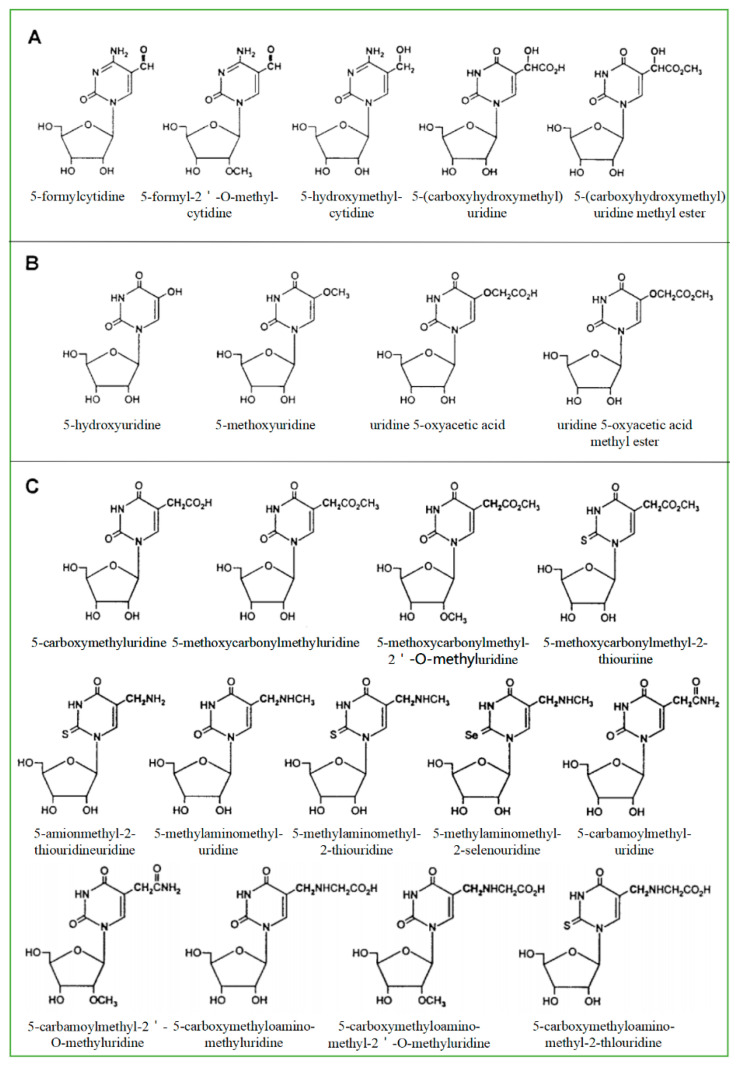
Modified pyrimidine nucleosides found in tRNA. They are divided into three subgroups according to possible factors: formylcytosine (**A**), hydroxyuridine (**B**), and carboxyuridine (**C**).

As a product of normal metabolism and obtaining from exogenous sources, living cells produce highly active ^−^OH, which is likely to cause a lot of the oxidative damage of biological macromolecules including RNAs. The most common source of ^−^OH in cells is the Fenton and Haber-Weiss reaction [24]. ^−^OH generated near RNA can readily modify RNA because they are so active that they cannot diffuse from where they are formed. Therefore, the modification caused by the hydroxyl group is the most diverse type of RNA damage. There are more than 20 distinct types of ^−^OH that destroy substrates [25]. Currently, 8-hydroxyguanosine (8-OHG) is the most common biomarker that can indicate RNA oxidation. The highly active hydroxyl radical first reacts with guanine to form C8-OH adducts and then produces 8-OHG, accompanied by the loss of an electron (e^−^) and proton (H^+^). According to the chemical structure of all known modified RNA nucleosides, they are divided into four categories base on the following chemical properties [24]: (1) The size of the modification group: small or large, like methyl- versus isopentenyl- groups. (2) The nature of basic substituents: simple or derivatives of other natural ingredients like amino acids and sugars and thio-threonine derivatives. (3) Type of modification: primary or secondary, such as adenine isoprenylation and Q or Y nucleoside side-chain substitution. (4) Synthetic pathway: enzymatic pathway (prenylation and methylation) or random pathway (hydroxylation or peroxidation). Among all known RNAs, tRNA is mainly responsible for carrying amino acids into ribosomes, synthesizing proteins under the guidance of mRNA, and can also participate in DNA synthesis by serving as reverse transcriptase primers. One of the structural characteristics of tRNA is that it contains more modified components, and most of the modified components in nucleic acids are found in tRNA [26]. The modified bases and nucleosides found in tRNA have the following characteristics: (1) modified bases and nucleosides tend to appear in many tRNA at position 34 (Swing position, when the anticodon of tRNA is paired with the codon of mRNA, the first two pairs strictly abide by the rule of base pairing, but the third pair of bases has a certain degree of freedom to “swing”); (2) it can produce pyrimidine residues, most of which are uridine derivatives; (3) it is easy to occur at position five outside the ring; (4) after modification, two uridine derivatives are generated in many cases (Figure 1). Based on their secondary reactions with formylcytosine (Figure 1A), hydroxyuridine (Figure 1B), and carboxyuridine (Figure 1C), these RNA-modified nucleosides can be further divided into three subgroups. In fact, oxidative damage-induced cleavage and fragmentation of tRNA can be observed in yeast and human cell lines [27]. Under oxidizing conditions, tRNA is cleaved at the anticodon loop into half molecules of 30–45 nucleotides in length [28,29], known as tRNA-derived stress-inducing fragments (tiRNAs) [30]. This conformational change of tRNA can be used as a biomarker to identify organ damage or reflect clinical prognosis [31]. In addition, tRNAs are an essential part of the translation mechanism. Oxidative damage to tRNA may lead to defects in codon-anticodon pairing or aminoacylation, which may lead to the production of incorrectly encoded proteins [8,21].

### 3.2. Different Forms of RNA Oxidative Damage

We have known for decades that poly (A)^+^mRNA accounts for only a small portion (about 1–2%) of the total cellular RNAs. RNA inside cells is primarily made up of rRNAs and tRNAs. Additionally, there are many different types of non-coding RNAs, such as microRNAs (miRNAs), small nucleolar RNAs (snoRNAs), and small nuclear RNAs (snRNAs) [32]. These non-coding RNAs play an essential role in mRNA splicing regulation, non-spliced RNA modification, and mRNA translation [33]. In regions affected by AD, which is the cytoplasm of hippocampal neurons, bound redox-active iron oxidizes rRNAs [34]. Oxidation of rRNA in the brains of patients with AD and mild cognitive impairment (MCI) is significantly increased, and oxidative damage also occurs in other types of cytoplasmic RNAs (such as tRNA and miRNA) in diseased tissues [35]. There are many forms of oxidative damage to RNA, which can affect the body in the following ways:

(1) Direct RNA strand breaks: Jacobs et al. found that the most common form of damage to RNA is the direct strand break [36]. The possible mechanism is the production of nucleic acid bases or their peroxyl groups, resulting in the removal of hydrogen atoms from adjacent ribose molecules, which in turn leads to a breakage of the RNA strand.
(2) Translation errors caused by oxidized RNA: Oxidized mRNA can induce translation errors, which can lead to premature termination of translation or degradation of peptide chains, resulting in short-chain polypeptides and protein variation [37]. In addition, oxidative changes of bases on mRNAs cause mismatches with the bases on tRNAs during translation and can result in protein variation.
(3) RNA oxidative damage can cause protein synthesis disorders: Experiments in rat primary nerve cell culture showed that the RNA in nerve cells is selectively oxidized when oxidative damage occurs. Generally, mRNA is more likely to be oxidized. During the transcription process, the presence of oxidized bases on the oxidized mRNA chain will cause transcription errors in the body, which will lead to the next translation error. Once an error occurs during translation, the expression of the corresponding protein will change. Ding et al. [38] demonstrated that once RNA is oxidized, protein synthesis in primary neurons and neural SH-SY5Y cells is significantly reduced. This decrease gradually increases with the extension of the oxidation time.

### 3.3. RNA Repair and Prevention of Oxidative Damage

In previous studies, it was believed that RNA only served as a messenger in the expression of genetic material, while ignoring the occurrence of oxidative damage to RNA may have important physiological and pathological effects [39]. Cells produce different repair mechanisms when dealing with different forms of RNA damage. Cells have evolved a variety of mRNA monitoring and control mechanisms to eliminate false transcripts, including nonsense-mediated mRNA decay (NMD), mRNA without stop codons, and degradation of translationally blocked mRNAs wait [40]. The oxidative modification of RNA is also affected by the RNA monitoring mechanism. Eukaryotic cells use NMD as the major mechanism to monitor RNA. Transcripts containing premature stop codons can be identified and degraded to avoid the accumulation of truncated protein products and reduce cytotoxicity [41]. Studies show that *E. coli* enzyme AlkB and human homologous enzyme hABH3 can directly reverse the alkylation damage of RNA by hydroxylating methyl groups on damaged RNA bases, and oxidized bases on RNA may be repaired [42].

## 4. Relationship Between RNA Oxidation and Neurodegenerative Disorders

Neurodegenerative Disorders are characterized by delayed onset and dysfunction of selective neurons. They are a type of irreversible neurological diseases caused by the loss of neuronal cells in the brain and spinal cord, including AD, PD, amyotrophic lateral sclerosis (ALS), and Huntington’s disease (HD) [43] (Table 1). The common clinical manifestation of these diseases is a decline in cognitive ability, presented as a patient’s ability to process, store, and extract information weakened gradually. Although they have different histopathological characteristics, they may share common cellular and molecular mechanisms [44]. Neurodegenerative disorders are common and age-related. For example, there are 65 AD patients and 9.5 PD patients per 1000 elderly people in the United States, while the annual incidence of ALS is 1.6 [45]. There is much evidence that oxidative damage is involved in the pathogenesis of neurodegenerative disorders, including AD and PD [46].

### 4.1. Early Events During Neurodegeneration

The dysfunction of nerve cells in specific areas caused by RNA oxidation is an important pathogenesis of neurodegenerative diseases (Table 2). Studies have confirmed that oxidative damage of neuronal RNA is closely related to cognitive deficits and occurs in the early stages of pathological changes in various neurodegenerative disorders [54]. Studies have found that excessive production of ROS, the main product of oxidative metabolism produced by the mitochondrial respiratory chain, may lead to mitochondrial damage [55]. Increased oxidative RNA is observed in the brain of patients with MCI after death, which is a transitional state between normal aging and dementia, and is also observed in different subjects with AD symptoms [56,57,58]. The sequential relationship between oxidative damage and pathological changes indicates that the oxidation of RNA in neurons precedes the formation of β-amyloid protein (A β) [59].

### 4.2. RNA Oxidation and Alzheimer’s Disease

**Figure 2:**
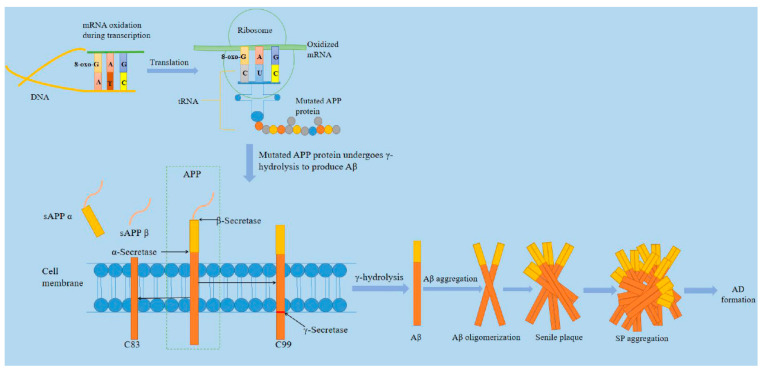
A brief mechanism of Alzheimer’s disease. The mRNA of amyloid precursor protein (APP) gene changes the process of gene transcription and translation due to the base mismatch produced by oxidation, which leads to the change of amino acid sequence of APP protein. Mutant APP protein produces more types and quantities of β-amyloid protein (A β) fragments through pathological β and γ hydrolysis pathways, and aggregates to form senile plaque (SP), to initiate the pathological process of AD [75].

Alzheimer’s disease, discovered by German psychiatrist and neuroanatomist Alois Alzheimer in 1907, is one of the main types of neurodegenerative disorders [64]. Most reports from the International Federation of Alzheimer’s disease shows that there are currently more than 35 million patients worldwide [65]. It is reported that the number of Americans aged 65 and older suffering from Alzheimer’s disease is about 5.8 million, which could rise to 13.8 million by the middle of this century, and official death certificate records show that the number of deaths from Alzheimer’s disease increased by 146.2% between 2000 and 2018, with 122,019 people dying of AD in 2018 alone [66]. AD is a neurodegenerative disease during which brain neurons produce progressive, irreversible loss, especially cortical and hippocampal neurons. The main clinical features are cognitive impairment, memory impairment, and personality change. The neuropathological changes are mainly characterized by extracellular senile plaque (SP), neurofibrillary tangles (NFT) formed by excessive phosphorylation of proteins in the brain cells, and the loss of a large number of neurons [67]. As the main component of senile plaques, Aβ is formed from amyloid precursor protein (APP) by co-cleaving with β- and γ-secretase [68]. In 2002, Hardy proposed the "Aβ cascade theory," which believed that the imbalance of Aβ production and clearance in brain tissue was the initial factor for the occurrence of AD [69,70]. As shown in Figure 2, Aβ is derived from APP and belongs to type Ⅰ transmembrane glycoprotein. Aβ sequence belongs to a segment of APP transmembrane region. Because its structure is dominated by the β sheet, it is called beta-amyloid. Aβ in senile plaques mainly exists in two forms of Aβ1-40 and Aβ1-42 [71]. Aβ undergoes the following five steps from production, aggregation, and exertion of neurotoxicity: APP gene→APP mRNA→APP protein→Aβ polypeptide→Aβ polypeptide aggregation [72]. We have known that after the guanine base on the RNA chain is oxidized to 8-oxoguanine (8-oxoG), it binds to cytosine C and adenine A on the DNA chain with the same efficiency during transcription. Once the oxidized guanine matches with A, the occurrence causes uracil U to be replaced by G (basically 8-oxoG) on the mRNA chain. The 8-oxoG carried on tRNA should be mismatched with A on mRNA when translating into proteins. Even if tRNA does not carry oxidizing bases, mismatching may be induced by 8-oxoG on mRNA strands. The types of oxygen acids carried by tRNA will be changed, resulting in disordered proteins [73]. Some researchers have found that 8-oxoG, the oxidation product of RNA, is significantly increased in the brain of patients with AD and is significantly related to the progression of the disease. The mRNA of APP gene changes the process of gene transcription and translation due to oxidation, which leads to changes in the amino acid sequence of APP protein. The mutant APP protein produces more types and quantities of Aβ fragments through pathological β- and γ-hydrolysis pathways, aggregating to form aging plaques, and initiates the pathological process of AD [74].

### 4.3. RNA Oxidation and Parkinson’s Disease 

PD, which was discovered by James Parkinson in 1817, was described as rest tremor, bradykinesia, muscular rigidity, and postural and gait impairment [76]. Since then, PD has been gradually defined as a typical neurodegenerative disease, characterized by the progressive degeneration of substantia nigra (SN) dopaminergic neurons, resulting in neuronal dysfunction and loss, which ultimately leads to exercise obstacle [77]. PD ranks second common neurodegenerative diseases after Alzheimer’s disease. Patients with PD will show clinical features such as bradykinesia, resting tremor, and rigidity, resulting in a significant decrease in quality of life [78]. During cell aging, one of the leading causes of cell mutagenesis and death is the accumulation of oxidative damage in cellular RNA. The oxidation of RNA leads to transcription errors and translation barriers, which in turn leads to a reduction in the synthesis of enzymes and proteins, and eventually cell death. It has been confirmed that RNA oxidative damage (mainly producing 8-oxoG) is accumulated in the nucleus and mitochondria of senescent cells [79]. In PD patients, this accumulation may increase dramatically. For example, it is reported that 8-oxoG is accumulated in the cytoplasm of SN dopamine neurons in PD patients, and in these neurons, *MTH1* with oxidized purine nucleoside triphosphatase activity is expressed [80]. During the loss of tyrosine hydroxylase (TH)-positive dopamine neurons induced by Phenyl-4-phenyl-1,2,3,6-tetrahydropyridine (MPTP), 8-OHG level rises in the RNA of striatum system cells [80]. All of this indicates that RNA oxidative damage is related to the loss of dopamine neurons [80]. In the cerebrospinal fluid of PD patients, the relationship between the level of 8-OHG and the course and severity of dementia also suggests that RNA oxidation may occur early in PD [62]. In order to study the extent and distribution of nucleic acid oxidative damage in fragile dopaminergic neurons, Zhang et al. [81] characterized the 8-hydroxyguanosine (8-OHG), a common product of nucleic acid oxidation, by immunohistochemistry, and found the immune response of 8-OHG in the cytoplasm is stronger than the nucleus. The test found that the oxidative damage of nucleic acid in PD patients occurs to a large extent in the cytoplasm, and both RNA and mitochondria are oxidation targets. These results indicate that oxidative damage to the cytoplasmic ribonucleic acid of SN neurons in PD patients is increased, and RNA oxidative damage may cause neurodegenerative diseases.

### 4.4. RNA Oxidation and Other Neurodegenerative Diseases 

In addition to AD and PD, HD and ALS are also common neurodegenerative diseases [82]. As a public health problem, these diseases affect tens of millions of people all over the world [83]. HD is an autosomal dominant inherited and eventually fatal neurodegenerative disease caused by the extension of the CAG chain encoding polyglutamine in the Huntington (HTT) gene and translated into mutant HTT (mtHTT) protein [84]. The clinical symptoms of HD are age-dependent, progressive motor dysfunction, mental disorders, and cognitive decline [85]. In the early stages of disease development, RNA oxidation may participate the transcription and translation of the CAG repeat of the first exon of the Htt gene, which in turn causes mutations in the Htt protein, resulting in neuronal dysfunction and striatum cell death and accelerated HD formation [86].

ALS is a neuromuscular disease among various neurodegenerative diseases, which usually occurs after the age of 50 and fatal respiratory paralysis occurs within 3–5 years after diagnosis, which is life-threatening [87,88]. In the process of ALS formation, RNA oxidation may change the structure of proteins, resulting in abnormal protein inclusion bodies gathering in the cytoplasm, and aggregates can, in turn, induce the production of ROS in cytoplasm and mitochondria, resulting in a harmful cycle in this way, thus promoting the formation of ALS [89,90].

To sum up, in patients with AD, PD, HD, ALS, and other neurodegenerative diseases, it is suggested that RNA is highly oxidized in the early stage of the illness before cell death, and this non-random and selective damage may affect the translation process [4,33,63]. For living organisms, once RNA is oxidized, the protective mechanism to reduce oxidized RNA is overwhelmed or loses its function, the accumulation of oxidized RNA will lead to the production of abnormal proteins, which is likely to lead to the onset of neurodegenerative diseases, so to rescue it is essential that RNA oxidizes and reduces the risk of related diseases [91]. Cell can maintain normal function and survive under stress conditions in order to reduce the level of RNA oxidation. At present, little is known about these mechanisms, but they can generally pass: repair of oxidized RNA; degradation of oxidized RNA; blocking oxidation incorporation of nucleotides into RNA; and other methods to reduce the harmful effects of RNA oxidation [92].

## 5. Discussion and Future Perspectives

With the rapid development of technology, people gradually realize that RNA is more susceptible to oxidative damage, and the oxidative damage of RNA is related to the early development of various neurodegenerative diseases such as AD, PD, and ALS [7]. This phenomenon may represent a common theme of the pathogenesis of neurodegenerative diseases, and it interferes with the normal translation process, promotes the synthesis of variant proteins, and subsequently initiates inappropriate cell fate pathways, eventually leading to various neurodegenerative diseases [4,63]. In this review, we emphasize that the appearance of RNA oxidative damage can suggest the occurrence of neurodegenerative diseases. Current research has confirmed that 8-OHG can be used as a marker of RNA oxidative damage to indicate the degree of RNA oxidative damage in the body, providing some reference information for understanding the course and treatment effect of neurodegenerative diseases such as AD and PD [24]. Understanding the important role of RNA oxidative damage in the pathogenesis of neurodegenerative diseases and the possible cellular therapeutic mechanisms may provide clues for the treatment strategies of neurodegenerative diseases in the future.

Oxidative damage to RNA can lead to interruption of the translation process and impaired protein synthesis. However, we are still in the preliminary stage of understanding the role of RNA oxidation in protein synthesis inhibition and cell death. We believe that the direction of further research can focus on the following issues. Under normal physiological conditions, whether oxidatively damaged RNA is degraded or repaired by the body and what protein is involved in the process is a very challenging question, and thus further research is needed [93,94]. It may be possible to identify proteins involved in this process by isolating repair/degradation complexes and applying protein microarrays or proteomics analysis. How to prevent oxidative damage to RNA through transgenic or pharmacological methods is also a good research direction. In order to better understand the functional role of RNA oxidation in the pathogenesis of various diseases, it is necessary to further study the above mechanisms.

In addition, recent research on RNA oxidation has a more interesting point: RNA oxidation is a feature of aging brain neurons, and it is more prominently observed in the early fragile neurons of age-related neurodegenerative diseases [95]. This suggests that the aging process may be closely related to RNA oxidation and many researchers have conducted preliminary research on this phenomenon. Comparative experimental studies on the brains of young and old mice [96] and studies on the brains of accelerated aging mice [97] suggest that the increase in 8-OHG in neurons of the brain is related to age. These findings further support RNA oxidation involved in the process of aging and neurodegenerative diseases. However, only a few studies have elaborated on this phenomenon. Continued in-depth understanding of the consequences of RNA oxidative damage and the underlying mechanism of aging will help provide better anti-aging methods in the future.

## Figures and Tables

**Table 1:** Summary of the classification of neurodegenerative disorders.

Diseases	Definition	Characteristics	References
AD	• AD is a progressive neurodegenerative disease characterized by generalized dementia.	• In patients, ROS levels are increased, endoplasmic reticulum protein folding and protein clearance are impaired, resulting in Tau protein accumulation. • In mouse model, peroxidase intervention can significantly improve cognitive and memory abilities and reduce plaque A deposition in the cerebral cortex and hippocampus.	[12,47,48]
PD	• PD is also a common neurodegenerative disease, its clinical features are static tremor and postural instability.	• 1-Methyl-4-phenyl pyridine ion (MPP +) accumulates in neuron mitochondria, inhibits the activity of mitochondrial respiratory chain complex I, increases ROS release, causes neuronal degeneration and necrosis, which eventually leads to the occurrence of PD.	[49,50,51]
HD	•HD is an inherited neurodegenerative disease, which is the fourth autosomal dominant hereditary disease. The main pathological changes were neuronal damage in basal ganglia and cerebral cortex, obvious atrophy of caudate nucleus, and enlargement of anterior horn of bilateral lateral ventricle. Cerebral cortex atrophy and whole brain atrophy can lead to dementia.	• Obstacles in the interaction between mitochondrial morphology and functionally stable proteins in HD patients are responsible for insufficient energy supply and neurodegeneration. • Animals with HD have reduced antioxidant capacity and impaired mitochondrial function, suggesting that oxidative stress may play an important role in the pathogenesis of HD.	[52,53]

**Table 2:** Review demonstrating that RNA oxidation plays a remarkable role in neurodegeneration.

Diseases	Role of Oxidative RNA	References
Alzheimer’s disease	• RNA oxidation is more pronounced in hippocampal neurons without neurofibrillary tangles. • RNA oxidation increases due to mutations in the presenilin-1 gene. • RNA oxidation is more prominent in the early stages of disease development.	[9,56,59]
Mild cognitive impairment	• RNA oxidation is increased in the brains of people with mild cognitive impairment, and these patients partially represent dementia.	[57]
Downsyndrome	• RNA oxidation occurs before the deposition of amyloid plaques in Down syndrome.	[60]
Subacute sclerosing panencephalitis	• RNA oxidation was observed in the early stages of the disease, and lipid peroxidation was observed in the case of longer disease duration.	[61]
Parkinson’s disease	• In the early stages, RNA oxidation is more prominent.	[62]
Amyotrophic lateral sclerosis	• In the early stages of symptoms, although motor neurons still look healthy, the level of RNA oxidation is significantly increased.	[63]
Cortical neuronal cultures oxidative insults	• RNA oxidation is an early event in the course of the disease, even before the death of neurons during neurodegeneration.	[33]
